# Portrait of French-speaking minorities with respect to vaccination against COVID-19

**DOI:** 10.14745/ccdr.v49i78a04

**Published:** 2023-08-01

**Authors:** Chloé Desjardins, Jennifer Lacroix Haraysm, Joseph Abdoulnour, Manon Denis-LeBlanc, Daniel Hubert, Salomon Fotsing, Diane Bouchard Lamothe, Sylvain Boet

**Affiliations:** 1 Francophone Affairs, Faculty of Medicine, University of Ottawa, Ottawa, ON; 2 Institut du Savoir Montfort, Ottawa, Ottawa, ON; 3 Department of Family Medicine, University of Ottawa, Ottawa, ON; 4 Faculty of Education, University of Ottawa, Ottawa, ON; 5 Department of Anesthesiology and Pain Medicine, University of Ottawa, Ottawa, ON; 6 Department of Innovation in Medical Education, University of Ottawa, Ottawa, ON; 7 Clinical Epidemiology Program, The Ottawa Hospital Research Institute, Ottawa, ON; 8 Keenan Research Centre at the Li Ka Shing Knowledge Institute, Toronto, ON

**Keywords:** vaccines, vaccine hesitancy, Francophone minorities, community survey

## Abstract

**Background:**

The coronavirus disease 2019 (COVID-19) vaccination campaign highlighted the requirement to better understand the needs of different populations. French-speaking minorities (FSMs) have greater difficulty accessing quality care in French, and this problem was exacerbated during the COVID-19 pandemic.

**Objective:**

The aim of this survey was to develop a descriptive portrait of the health needs of FSMs in relation to the COVID-19 vaccination campaign by describing their vaccination status, attitudes and beliefs compared with English-speaking majorities.

**Methods:**

A survey was conducted among eligible participants using convenience sampling. Data measurement includes a descriptive statistical comparison using analysis of the variance, univariate logistic regressions and a two-proportions z-test.

**Results:**

Of the 1,505 respondents (554 FSMs vs. 951 English speakers), the FSMs have an average age of 51.4 years and 89.2% are Canadian citizens. Vaccination of children was preponderant among English speakers (74.2% vs. 86.3%), including against COVID-19 (58.6% vs. 73.9%). A higher proportion of FSMs had gotten vaccinated in order to obtain a vaccine passport (39% vs. 29.3%). Among the unvaccinated, FSMs were more likely to question the efficacy of vaccines (60% vs. 36.4%). Canadian citizen FSMs with higher education could be divided in relation to the vaccine regimen.

**Conclusion:**

This survey revealed differences between FSMs and the English-speaking majority in their perceptions of vaccine efficacy, particularly vaccination of children, and a polarization of attitudes/beliefs among FSMs according to certain sociodemographic factors.

## Introduction

The coronavirus disease 2019 (COVID-19) vaccination campaign highlighted the requirement to better understand the needs of different Canadian populations during a pandemic. The lack of data on the needs of linguistic minorities ((1–3)) had a significant impact on vaccine uptake and trust in healthcare institutions ((4,5)).

French-speaking minorities (FSMs) have greater difficulty accessing quality care in French ((6–12)), which is one of the problems exacerbated during a pandemic ((8,12,13)). However, vaccine uptake is influenced by multiple factors linked to the sociocultural context, including values, morality, accessibility and therapeutic experience, requiring adapted medical practices ((14–17)). This study is necessary to fill the knowledge gap on the subject and improve the active offer.

Given the fragmented nature of Canadian Francophonie ((18)), it is difficult to establish an overall picture of the needs of FSMs based on up-to-date evidence. An existing survey ((19)) explores some relevant areas, but does not provide a breakdown by language, at least not in publicly available data. This survey, carried out between May 1 and June 30, 2022, aims to describe the health needs of FSMs in relation to the COVID-19 vaccination campaign through the lens of vaccination status, attitudes and beliefs, and provides for a comparison with English-speaking majorities.

## Methods

This article was written according to the guidelines of Improving the Quality of Web Surveys: The Checklist for Reporting Results of Internet E-Surveys (CHERRIES) ((20)).

### Population, time and place

The survey was conducted over an eight-week period ending on June 30, 2022, among FSMs and English speakers outside Québec, Canada. The study defines FSMs as residents outside Québec whose preferred language is French, and Anglophones as residents outside Québec whose preferred language is English. Given the rapid evolution of the pandemic, convenience sampling was used.

### Link to the research objective

The descriptive portrait of FSMs vis-à-vis the COVID-19 vaccination campaign includes the collection of sociodemographic data, vaccination status, attitudes and beliefs.

### Development of the survey questionnaire

The questionnaire (**Supplemental material**, **Survey**) was designed by the research team based on a validated survey ((19)) by Statistics Canada. To meet the requirements of the study, questions dealing with language, attitudes and beliefs were added before conducting a pilot study with 30 participants drawn from the mailing list of Léger Marketing Inc.

### Sampling technique

Participants were recruited primarily via the sampling strategy, the mailing list of Léger Marketing Inc. and Canadian Francophone organizations (Supplemental material, **Survey invitation letter**). The sample was created taking into account the response rates for each age category and the quotas required to obtain a representative sample. Representative quotas were established for age, gender and province. The sample was sent out strategically to ensure representativeness. For example, attention was focused on the 18 to 24 age group, as these respondents are generally harder to reach, while less attention was paid to the 65+ age group, as they are conversely much easier to reach. This required constant attention to the quotas defined in the survey platform, while ensuring random selection. An invitation letter, a consent form and the questionnaire were distributed to those who met the inclusion criteria.

### Informed consent

The study was approved by the University of Ottawa Research Ethics Board (H-02-22-7648). A consent form had to be completed by participants prior to conducting the survey.

### Optimizing response rates

The survey was made available on FocusVision Decipher (Forsta, 2022) and on the LEO mobile app (Léger Marketing Inc., 2020), in addition to being widely distributed via the social networks of the University of Ottawa Faculty of Medicine’s Francophone Affairs. Participants were invited to share the survey, allowing snowball sampling to be used to optimize the response rate.

### Measurement

Data measurement was carried out in accordance with two research questions designed to identify 1) the vaccination status, attitudes and beliefs of FSMs compared with English speakers, and 2) the sociodemographic characteristics of FSMs in relation to vaccination status, attitudes and beliefs.

Sociodemographic data includes: province/territory of residence, age, gender, income, education, marital status, ethnicity, citizenship and health status. Vaccination status includes COVID-19 vaccine doses, willingness to follow the recommended vaccine regimen, and vaccination of children (ages 5 to 11 years). Attitudes include reasons for uptake and hesitancy, as well as trusted sources of information. Beliefs include vaccine safety, perceived risks and efficacy, health practices and social responsibility.

## Analysis

Descriptive statistics were calculated and analyzed using SPSS (version 22.0). Continuous variables were presented as means and standard deviations, and categorical variables as totals and/or percentages. Analyses of variance (ANOVA) were performed to examine significant differences in continuous variables. Univariate logistic regressions were performed to determine the associations between FSMs and English speakers, and also sociodemographic variables with vaccination status and belief. The findings are presented as odds ratios (OR) with 95% confidence intervals (CI), as well as the likelihood chi-squared statistic. A two-proportions z-test was performed for multiple-response questions to compare proportions between groups; the Bonferroni correction was used for multiple comparisons. A *p*-value of less than 0.05 indicates a statistically significant difference.

## Findings

The sample comprised 1,505 participants: 554 FSMs and 951 English speakers. The findings include a 100% response rate for each participant, giving n=554 (FSMs) and n=951 (English speakers). The sociodemographic data are presented below (**Table 1**).

**Table 1:** Sociodemographic characteristics of French-speaking minority participants and English-speaking participants

Characteristics	% FSM (n=554)	% English speakers (n=951)
**Age (years)**		
Mean; standard deviation	51.4; 16.9	48.1; 17.4
Median	53.0	47.0
18–24	4.7	11.1
25–34	17.0	15.3
35–44	14.6	17.0
45–54	17.5	20.1
55–64	20.9	17.5
65–74	17.5	9.3
≥75	7.8	9.6
**Gender**		
Female	61.2	50.2
Male	38.8	49.8
Other	0.0	0.0
Prefer not to answer	0.0	0.0
**Province**		
Ontario	47.1	50.4
New Brunswick	33.4	2.4
British Columbia	6.5	17.8
Alberta	6.5	14.2
Manitoba	2.7	4.9
Saskatchewan	1.4	4.1
Nova Scotia	1.4	3.7
Nunavut	0.4	0.0
Newfoundland and Labrador	0.2	2.1
Prince Edward Island	0.2	0.4
Yukon	0.2	0.0
**Income**		
≤$30,000	12.4	13.2
$30,000 to $60,000	23.4	23.6
$60,000 to $90,000	20.7	22.0
$90,000 to $120,000	17.4	17.9
$120,000 to $150,000	11.3	9.8
>$150,000	14.8	13.5
**Education**		
Less than a high school diploma or equivalent	3.1	1.2
High school diploma or certificate of equivalence	15.6	18.2
Trade certificate or diploma	5.6	6.9
College, CEGEP or other non-university certificate or diploma	20.0	22.4
University certificate or diploma below bachelor level	5.1	6.8
Bachelor’s degree	30.0	30.5
University certificate, diploma or degree above bachelor level	20.5	14.0
**Marital status**		
Single	23.4	24.1
Couple	49.5	34.9
Family	27.2	40.9
**Indigenous status**		
North American First Nation	1.3	2.1
Métis	2.5	2.0
Inuk (Inuit)	0.0	0.3
**Ethnicity**		
Arab	0.9	1.4
Southeast Asian	1.6	0.5
West Asian	0.7	0.2
Caucasian	70.8	91.5
Chinese	8.8	1.3
Korean	0.6	0.0
Japanese	0.5	0.0
Latin American	1.5	0.5
African American	1.8	2.5
Filipino	1.3	0.0
South Asian	6.6	0.5
Other	4.7	1.6
**Citizenship status**		
Canadian citizen by birth	89.2	77.2
Canadian citizen by naturalization	7.6	18.6
Permanent resident	2.5	2.7
None	0.7	1.5
**State of health**		
Obesity	9.4	9.2
Heart and/or vessel disease	4.7	4.7
Diabetes	10.1	6.3
Liver disease	0.7	0.4
Chronic kidney disease	0.0	0.7
Alzheimer’s disease	0.2	0.0
Immunodeficiency	3.3	3.3
Lung disease	7.2	6.7
None of these health problems	64.3	68.7

Abbreviation: FSM, French-speaking minority

## Vaccination status

### Differences between French-speaking minorities and English speakers

According to the univariate regression values, FSMs were less willing to have their children vaccinated against preventable diseases (74.2% vs. 86.3%) (χ^2^[1, N=440]=7.069, *p*=0.008; OR=0.455 [95% CI: 0.259–0.799]), against COVID-19 (58.6% vs. 73.9%) (χ^2^[1, N=436]=7.531, *p*=0.006; OR=0.500 [95% CI: 0.306–0.815]) or to follow the recommended vaccine regimen (0.0% vs. 22.0%) (χ^2^[3, N=126]=16.879, *p*=0.001) (**Table 2**).

**Table 2:** Vaccination status among French-speaking minorities and English speakers

Vaccination status	% FSM (n=554)	% English speakers (n=951)	Likelihood chi-squared	Approx. sig. (bilateral)^a^	OR	95% CI	
**Adult vaccinated against COVID-19**							
Yes	93.60	91.80	1.763	0.184	0.756	0.500	1.144
No	6.40	8.20			N/A	N/A	N/A
**COVID-19 vaccination doses**							
1 dose	0.80	1.30	5.758	0.124	0.472	0.144	1.549
2 doses	19.50	23.50			0.640	0.429	0.953
3 doses	66.70	65.20			0.790	0.559	1.116
4 doses	13.00	10.10			N/A	N/A	N/A
**Plausibility of following the recommended full vaccine regimen (vaccinated adult)**							
Very likely	62.20	62.60	2.463	0.482	0.881	0.559	1.390
Somewhat likely	20.10	22.50			0.792	0.483	1.300
Unlikely	11.10	9.10			1.082	0.623	1.879
Very unlikely	6.60	5.90			N/A	N/A	N/A
**Plausibility of following the recommended full vaccine regimen (unvaccinated adult)**							
Very likely	2.90	6.40	4.523	0.210	0.354	0.039	3.194
Somewhat likely	5.70	17.90			0.253	0.053	1.200
Unlikely	17.10	16.70			0.817	0.277	2.405
Very unlikely	74.30	59.00			N/A	N/A	N/A
**Previous vaccination for children (against other diseases)**							
Yes	74.20	86.30	7.069	0.008	0.455	0.259	0.799
No	25.80	13.70			N/A	N/A	N/A
**Children vaccinated against COVID-19**							
Yes	58.60	73.90	7.531	0.006	0.500	0.306	0.815
No	41.40	26.10			N/A	N/A	N/A
**COVID-19 vaccination doses**							
1 dose	29.20	17.20	3.382	0.184	2.064	0.784	5.433
2 doses	54.20	62.60			1.053	0.446	2.486
3 doses	16.70	20.30			N/A	N/A	N/A
**Plausibility of following the recommended full vaccine regimen (children)**							
Very likely	0.0	22.0	16.879	0.001	6.84E-10	6.84E-10	6.84E-10
Somewhat likely	28.6	34.10			0.473	0.180	1.247
Unlikely	28.6	19.8			0.815	0.296	2.246
Very unlikely	42.9	24.2			N/A	N/A	N/A

Abbreviations: CI, confidence interval; COVID-19, coronavirus disease 2019; FSM, French-speaking minority; N/A, not applicable; OR, odds ratio

^a^ Approx. sig. (bilateral) is a *p*-value of less than 0.05 for univariate analyses is considered significant

### Differences according to sociodemographic data

Compared with those born outside the country, Canadian-born FSMs are more inclined to not follow the recommended vaccine regimen (85.2% vs. 37.5%) (χ^2^[3, N=35]=10.714, *p*=0.013; OR=7.667 [95% CI: 1.035–56.770]), but have more doses (67.7% and 13.6% vs. 56.9% and 7.8%) (χ^2^[3, N=513]=9.848, *p*=0.020; OR=15.750 [95% CI: 1.736–142.882]). Among those, individuals with a college/certificate education are less inclined to agree with the vaccine regimen compared with those with a higher education (52.7% vs. 75.7%) (χ^2^[9, N=509]=22.968, *p*=0.006; OR=0.313 [95% CI: 0.109–0.903]). More FSMs are vaccinated in Ontario (96.2% vs. 86.2% [West] and 93.8% [Atlantic]) (χ^2^[2, N=547]=10.317, *p*=0.017; OR=4.012 [95% CI: 1.695–9.497]) receive more doses compared with other regions (20% vs. 8.6% [West] and 5.6% [Atlantic]) (χ^2^[6, N=511]=43.713, *p*<0.001). Men (18.9% vs. 9.3%, women) (χ^2^[3, N=514]=14.229, *p*=0.003; OR=2.044 [95% CI: 1.203–3.471]) and older individuals (52.2 ± 16.1 and 68.8 ± 11.2 years vs. 40.8 ± 18.3 and 40.9 ± 12.2 years; F(3, 510)=46.58, *p*<0.001) more often had 3–4 doses. Among FSMs with vaccinated children, a high income was preponderant (87% [>$120,000] vs. 56.8% [$60,000 to $120,000] vs. 34.6% [<$60,000]) (χ^2^[2, N=86]=14.963, *p*=0.001; OR=12.593 [95% CI: 2.931–54.107]).

### Attitudes

### Differences between French-speaking minorities and English speakers

There are two significant differences: a greater proportion of FSMs had gotten vaccinated to obtain the vaccine passport (39% vs. 29.3%, *p*<0.001); among the unvaccinated, more FSMs questioned the efficacy of the COVID-19 vaccine (60.0% vs. 36.4%, *p*=0.019) (**Table 3**).

**Table 3:** Vaccination attitudes between French-speaking minorities and English speakers

Vaccination attitudes	FSMs		English speakers		Statistical z-test^a^	*p*-value
	n	%	n	%		
**Reasons for vaccination (vaccinated adult)^b^**						
Vaccination is mandated by my workplace	112	21.7%	163	18.8%	−1.34	0.1811
Vaccination passport	201	39.0%	254	29.3%	−3.72	0.0002
I want to protect myself against serious illness	395	76.7%	686	79.0%	−1.02	0.3099
Return to normal life	275	53.4%	433	49.9%	−1.26	0.2064
I want to protect others	329	63.9%	574	66.1%	−0.85	0.3964
Leisure	179	34.8%	288	33.2%	−0.60	0.5487
Other	14	2.7%	22	2.5%	−0.21	0.8355
**Reasons for vaccine hesitancy (unvaccinated adult)^c^**						
The vaccine is not recommended for me	5	14.3%	7	9.1%	−0.83	0.4088
I do not have the necessary information to make a decision	4	11.4%	8	10.4%	−0.17	0.8688
I know too many people who have had side effects	12	34.3%	32	41.6%	−0.73	0.4642
I’m afraid	5	14.3%	9	11.7%	−0.39	0.6994
I am not at a great risk of contracting COVID-19	9	25.7%	17	22.1%	−0.42	0.6720
If I get COVID-19, I won’t be very sick	6	17.1%	17	22.1%	0.60	1.4517
We do not know the long-term side effects	22	62.9%	44	57.1%	−0.57	0.5681
I don’t know who to believe	3	8.6%	8	10.4%	−0.30	0.7640
I don’t know how, when or where to get vaccinated	0^d^	0.0%	1	1.3%	N/A^d^	N/A^d^
I should be given a choice	18	51.4%	36	46.8%	−0.46	0.6456
There was a problem with the appointment	0^d^	0.0%	2	2.6%	N/A^d^	N/A^d^
I didn’t have time	0^d^	0.0%	4	5.2%	N/A^d^	N/A^d^
I’ve already had COVID-19	3	8.6%	15	19.5%	−1.46	0.1446
I don’t want to get vaccinated at this time	14	40.0%	25	32.5%	−0.78	0.4370
In general, I don’t believe in vaccines	4	11.4%	10	13.0%	−0.23	0.8169
The vaccine I want is not available or has not been offered to me	0^d^	0.0%	2	2.6%	N/A^d^	N/A^d^
I don’t trust the vaccine offered to me	10	28.6%	20	26.0%	−0.29	0.7731
I don’t trust the health system	5	14.3%	10	13.0%	−0.19	0.8513
Cultural, philosophical or religious reasons	5	14.3%	7	9.1%	−0.83	0.4088
I’m pregnant or plan to become pregnant	1	2.9%	3	3.9%	−0.28	0.7833
I’m not sure that vaccines against COVID-19 are effective	21	60.0%	28	36.4%	−2.34	0.0194
Other	1	2.9%	10	13.0%	−1.67	0.0947
**Reasons for hesitancy concerning vaccination of children^e^**						
The vaccine is not recommended for them	7	20.0%	29	32.2%	−1.35	0.1754
I do not have the necessary information to make a decision	8	22.9%	11	12.2%	−1.49	0.1370
I know too many people who have had side effects	5	14.3%	14	15.6%	−0.18	0.8591
I’m afraid and/or my children are afraid	2	5.7%	6	6.7%	−0.20	0.8451
My children are not at high risk of contracting COVID-19	4	11.4%	11	12.2%	−0.12	0.9024
If they contract COVID-19, my children won’t be very sick	8	22.9%	10	11.1%	−1.68	0.0931
We do not know the long-term side effects of the vaccine that was offered to me for them	11	31.4%	27	30.0%	−0.16	0.8761
I don’t know who to believe	3	8.6%	3	3.3%	−1.23	0.2187
I don’t know how, when or where to get my children vaccinated	0^d^	0.0%	1	1.1%	N/A^d^	N/A^d^
I should be given a choice	8	22.9%	16	17.8%	−0.65	0.5174
There was a problem with the appointment	1	2.9%	2	2.2%	−0.21	0.8350
I didn’t have time	2	5.7%	2	2.2%	−1.00	0.3192
They’ve already had COVID-19	6	17.1%	10	11.1%	−0.91	0.3648
I don’t want my children to get vaccinated at this time	5	14.3%	19	21.1%	−0.87	0.3844
In general, I don’t believe in vaccines	0^d^	0.0%	6	6.7%	N/A^d^	N/A^d^
The vaccine I want for my children is not available or has not been offered to me	1	2.9%	3	3.3%	−0.14	0.8920
I don’t trust the vaccine offered to me	4	11.4%	10	11.1%	−0.05	0.9597
I don’t trust the health system because of a bad experience	3	8.6%	5	5.6%	−0.62	0.5362
Cultural, philosophical or religious reasons	0^d^	0.0%	3	3.3%	N/A^d^	N/A^d^
I’m not sure that vaccines against COVID-19 are effective	5	14.3%	21	23.3%	−1.12	0.2631
In general, the risks associated with vaccines are greater than the benefits	6	17.1%	15	16.7%	−0.06	0.9490
Other	0^d^	0.0%	4	4.4%	N/A^d^	N/A^d^
**Trusted sources of information on COVID-19 vaccination^f^**						
Friends, family members or acquaintances	51	9.3%	132	13.9%	−2.64	0.008
My physician	379	69.0%	657	69.4%	−0.14	0.890
My pharmacist	238	43.4%	380	40.1%	−1.23	0.220
Other healthcare professionals (e.g. nurses)	228	41.5%	439	46.4%	−1.82	0.069
Community leaders	17	3.1%	35	3.7%	−0.61	0.540
Politicians	24	4.4%	18	1.9%	−2.80	0.005
Social media	23	4.2%	26	2.7%	−1.52	0.129
Alternative medicine professionals	32	5.8%	48	5.1%	−0.63	0.527
Public health authorities	335	61.0%	529	55.9%	−1.95	0.051
Health scientists and researchers	352	64.1%	593	62.6%	−0.58	0.561
World Health Organization (WHO)	267	48.6%	437	46.1%	−0.93	0.351
Pharmaceutical companies	24	4.4%	70	7.4%	−2.34	0.020
Other	29	5.3%	59	6.2%	−0.75	0.451
**Means of validating COVID-19 vaccination information^g^**						
Confirm with other sources	338	61.6%	558	59.1%	−0.94	0.3481
Click on the link to read the full article	230	41.9%	461	48.8%	−2.59	0.0095
Check the date of the information	204	37.2%	354	37.5%	−0.13	0.8949
Check the number of likes or shares	6	1.1%	29	3.1%	−2.47	0.0134
Research the author or source	242	44.1%	407	43.1%	−0.36	0.7154
Read the comments or take note of the discussions on the subject	93	16.9%	164	17.4%	−0.21	0.8300
Consult friends and family	59	10.7%	142	15.0%	−2.33	0.0196
Check the credibility of the URL	203	37.0%	339	35.9%	−0.41	0.6785
Other	60	10.9%	86	9.1%	1.15	1.7482

Abbreviations: COVID-19, coronavirus disease 2019; FSM, French-speaking minority; N/A, not applicable

^a^ Statistical z-test results are based on bilateral tests with a significance level of 0.05. The tests are adjusted for all pairwise comparisons within a row of each innermost sub-table, using the Bonferroni correction

^b^ Total N for FSMs=35 and for English speaking=77

^c^ This category is not used in the comparisons as its proportion of columns is equal to zero

^d^ Total N for FSMs=515 and for English speaking=868

^e^ Total N for FSMs=35 and for English speaking=90

^f^ Total N for FSMs=549 and for English speaking=947

^g^ Total N for FSMs=549 and for English speaking=944

### Differences according to sociodemographic data

French-speaking minorities who are Canadian citizens by birth are mainly vaccinated for a return to normal life (55% vs. 39%, *p*=0.034) and protection against serious illness (79% vs. 59%, *p*=0.002). To obtain information on COVID-19, they mainly consulted family and friends (10% vs. 20%, *p*=0.015), pharmacists (45% vs. 30%, *p*=0.026) and public health authorities (63% vs. 47%, *p*=0.016). Ontarians are more confident in the safety and efficacy of vaccines/health measures (58.1% vs. 38.9% [West] and 42.7% [Atlantic]) (χ^2^[6, N=545]=19.141, *p*=0.004; OR=1.829 [95% CI: 0.786–4.255]). This confidence is also preponderant among men (58.4% vs. 43.4%, women) (χ^2^[3, N=548]=12.337, *p*=0.006; OR=1.724 [95% CI: 0.804–3.695]) who are more willing to get vaccinated to protect themselves against serious illness (83% vs. 72.6%, *p*<0.001). The higher the level of education, the more likely it was that article publication dates would be consulted to validate information (40% vs. 24%, *p*=0.008) and that scientific professionals would be regarded with confidence (76% vs. 56%, *p*<0.001).

### Beliefs

### Differences between French-speaking minorities and English speakers

FSMs frequently disagreed with the efficacy of herd immunity (**Table 4**).

**Table 4:** Vaccination beliefs among French-speaking minorities and English speakers

Vaccination beliefs	% FSM (n=554)	% English speakers (n=951)	Likelihood chi-squared	Approx. sig. (bilateral)	OR	95% CI	
**Vaccines are safe despite the risks**							
Strongly agree	52.00	51.40	5.561	0.135	3.009	1.023	8.854
Agree	40.60	39.70			2.971	1.114	7.923
Disagree	4.90	5.60			1.876	0.692	5.084
Strongly disagree	2.50	3.30			N/A	N/A	N/A
**COVID-19 vaccines are safe, despite the risks**							
Strongly agree	49.30	48.50	6.656	0.084	0.290	0.089	0.943
Agree	36.70	36.90			0.258	0.090	0.743
Disagree	8.00	9.00			0.342	0.134	0.875
Strongly disagree	6.00	5.70			N/A	N/A	N/A
**I distrust COVID-19 vaccines because they were developed too quickly**							
Strongly agree	10.30	9.60	1.981	0.576	0.692	0.366	1.310
Agree	15.90	16.40			0.763	0.468	1.245
Disagree	39.80	38.60			0.816	0.588	1.134
Strongly disagree	34.00	35.40			N/A	N/A	N/A
**By getting the COVID-19 vaccine, I am protecting myself against severe forms of this disease**							
Strongly agree	52.60	50.30	3.161	0.367	1.614	0.622	4.188
Agree	35.60	36.70			1.251	0.501	3.124
Disagree	7.10	7.40			1.556	0.642	3.772
Strongly disagree	4.70	5.60			N/A	N/A	N/A
**Physical distancing, frequent hand washing and wearing a mask are effective methods of slowing the spread of COVID-19**							
Strongly agree	58.00	56.10	3.332	0.343	0.734	0.295	1.828
Agree	34.60	35.60			0.616	0.250	1.514
Disagree	4.50	6.20			0.517	0.197	1.353
Strongly disagree	2.90	2.10			N/A	N/A	N/A
**Physical distancing, frequent hand washing and wearing a mask are enough to protect me against COVID-19**							
Strongly agree	13.60	11.60	1.311	0.727	0.853	0.537	1.356
Agree	28.50	29.50			0.795	0.529	1.196
Disagree	43.20	43.30			0.896	0.625	1.284
Strongly disagree	14.70	15.70			N/A	N/A	N/A
**Only those at risk of becoming seriously ill due to COVID-19 need to be vaccinated**							
Strongly agree	6.90	6.00	3.537	0.316	0.822	0.469	1.443
Agree	12.90	12.20			1.012	0.613	1.670
Disagree	36.70	35.40			0.771	0.550	1.080
Strongly disagree	43.60	46.40			N/A	N/A	N/A
**By getting vaccinated against COVID-19, I’m helping to protect the health of others in my community**							
Strongly agree	57.50	56.10	3.842	0.279	1.862	0.817	4.244
Agree	30.50	29.20			1.564	0.701	3.490
Disagree	6.50	8.60			1.032	0.464	2.297
Strongly disagree	5.50	6.00			N/A	N/A	N/A
**I prefer to develop immunity to COVID-19 by catching the disease than through the vaccination**							
Strongly agree	9.40	7.10	48.820	0.000	5.716	2.997	10.901
Agree	15.60	14.70			3.693	2.207	6.181
Disagree	40.60	29.30			2.918	2.060	4.134
Strongly disagree	34.40	48.90			N/A	N/A	N/A
**Those who have already had COVID-19 do not need to get vaccinated**							
Strongly agree	5.60	6.50	13.088	0.004	0.522	0.253	1.077
Agree	12.00	12.80			0.961	0.560	1.647
Disagree	49.00	39.00			1.489	1.079	2.055
Strongly disagree	33.40	41.70			N/A	N/A	N/A

Abbreviations: CI, confidence interval; COVID-19, coronavirus disease 2019; FSM, French-speaking minority; N/A, not applicable; OR, odds ratio

### Differences according to sociodemographic data

French-speaking minorities with high incomes, >$120,000, were not wary of the rapid development of the vaccines (47.2% [>$120,000] vs. 32.2% [$60,000 to $120,000] and 25.0% [<$60,000]) (χ^2^[6, N=546]=33.064, *p*<0.001; OR=6.381 [95% CI: 2.454–16.592]), did not believe in the stand-alone efficacy of physical distancing (21.7% [>$120,000] vs. 12.5% [$60,000 to $120,000] vs. 11.9% [<$60,000]) (χ^2^[6, N=544]=15.805, *p*=0.015; OR=3.836 [95% CI: 1.671–8.805]), or herd immunity (46.8% [>$120,000] vs. 30.8% [$60,000 to $120,000] vs. 29.1% [<$60,000]) (χ^2^[6, N=545]=20.787, *p*=0.002; OR=5.789 [95% CI: 2.080–16.112]) and that a previous diagnosis would result in less serious illness (42.6% [>$120,000] vs. 30.9% [$60,000 to $120,000] vs. 29.1% [<$60,000]) (χ^2^[6, N=544]=15.185, *p*=0.019; OR=5.965 [95% CI: 1,659–21,449]).

## Discussion

### Summary of key findings

The survey highlights three findings of interest: a polarization of attitudes/beliefs according to citizenship and education, vaccine uptake for a return to normal, and significant hesitancy concerning vaccination of children.

### Comparative analysis

Compared with English speakers, FSMs show a polarization of attitudes/beliefs according to certain sociodemographic characteristics. Among FSMs, Canadian-born citizens with a higher education were more likely to completely disagree or agree with the recommended vaccine regimen. This trend is noted by other studies in high-income countries ((17)). The literature indicates that mixed attitudes may stem from inconsistent information from official sources ((21–24)), becoming a risk to communication and patient disregard for medical care ((25)).

According to the literature, the prospect of a “return to normal” is strong motivation for vaccine uptake ((4,21)). Although FSMs generally doubted its efficacy, they mainly got vaccinated to obtain the vaccine passport and to protect themselves against serious illness, especially in the case of men. Given the inconsistency of information, also felt among healthcare professionals ((25)), FSMs were not always able to count on the news and relied on the recommendations of government agencies, promising a return to normality thanks to vaccination ((24,26)).

Although FSMs are often described as an older population ((7,27)), this survey was designed to be representative of all FSM generations. Despite the low representation of French-speaking parents with young children, vaccination hesitancy for children is of particular interest. Vaccine hesitancy (COVID-19 and other diseases) for children is more pronounced among FSMs, who are less likely to follow the vaccine regimen, unless they have a high income. In a broader context, the efficacy of COVID-19 vaccines in children has been widely disputed in literature ((17,28)).

The problem of childhood vaccination, which existed prior to the emergence of COVID-19 ((17)) and led to parental vaccine hesitancy during this pandemic ((28)), could be caused by sub-optimal physician-patient communication ((4,29)). The finding of this study could indicate greater inaccessibility for linguistic minorities. We hypothesize that the current shortage of family physicians in rural and urban settings ((30,31)), and by extension a lack of accessibility to bilingual health professionals, could contribute to an exacerbation of the problem of vaccination of children during a health crisis. Vaccination of children and parental hesitancy should be the subject of further research to pursue this line of thought and optimize access to care.

### Strengths and weaknesses

Considering the rapid evolution of the virus and of health recommendations, the study has some conceptual and methodological limitations. Media saturation and collective exhaustion made participation less appealing and influenced the sampling technique that was selected, resulting in a sampling bias caused by a convenience sample. Despite the strategy employed by Léger Marketing Inc., it is difficult to ensure the representativeness of FSMs and English speakers, as well as the potential for statistical generalization of the findings. Furthermore, the survey presents a portrait of FSMs for a given period, rather than according to a specific situation during the pandemic. The time elapsed between the data collection period and the comparative analysis must also be considered a bias for the representativeness of the findings. Despite this, the study met its objective and thus contributed to the active offering of French-language health services.

### Impact

This survey provides health professionals with the relevant information they need to tailor their communication with patients who are faced with a vaccination choice. The findings also point to the need for new studies establishing a portrait of FSMs in order to better address their vaccine needs.

### Next steps

By filling the knowledge gap regarding vaccination against COVID-19, this data could help improve access to information and, consequently, help adapt the training of health professionals for a therapeutic alliance based on trust.

## Conclusion

Although difficult to generalize, this survey did reveal significant differences between FSMs and English speakers in their perceptions of vaccine efficacy, particularly vaccination of children, as well as a polarization of the attitudes/beliefs of FSMs according to certain sociodemographic factors. The findings imply a requirement to better understand the overall needs of FSMs in order to improve access to information and care in French.

## Supplemental material

These documents can be accessed on the Supplemental material file
Survey, data collection tool
Survey invitation letter and distribution list
